# Supplementary material for: Post-discharge tobacco abstinence in a Mumbai hospital after implementation of tobacco cessation counseling: A pragmatic evaluation of the LifeFirst program
Source: PLoS One. 2024 Nov 12;19(11):e0312319. doi: 10.1371/journal.pone.0312319 (PMC11556754; doi:10.1371/journal.pone.0312319)
Supplement: S1 File — (PDF) [file pone.0312319.s001.pdf]

SURVEY FORM

Project title: Assessing and measuring tobacco use patterns among inpatients at the Prince Aly Khan Hospital

BASELINE SURVEY

<table>
<tr><td>UHID</td><td colspan="2">Date of admission:</td><td colspan="3">Date of interview:</td><td>Time started:</td></tr>
<tr><td colspan="5">Last name, first name:</td><td>Date of birth:</td><td>Gender: M/F</td></tr>
<tr><td colspan="2">Primary Language (language used by respondent for most of time):</td><td colspan="3">Indian National: Yes/No</td><td colspan="2">Telephone:</td></tr>
<tr><td colspan="5"></td><td colspan="2">Alt Tel:</td></tr>
<tr><td colspan="4">Place of usual residence (City/town/village)
______________________</td><td>Consultant:</td><td colspan="2">Specialty</td></tr>
<tr><td colspan="3">Instruction for investigators – (a) code it from the medical records only. (b) Do not share the diagnosis with the patient or relative.</td><td colspan="4">Medical/Surgical History:
☐ Cardiovascular disease
☐ Malignancy, list site:________________________
(For example, lung, head and neck, or stomach)
☐ Cerebrovascular disease/stroke
☐ Chronic lung disease
☐ HTN
☐ DM
☐ Other: ____</td></tr>
<tr><td colspan="3">Discharge diagnosis:</td><td colspan="4"></td></tr>
</table>

Note: To be filled for only for current tobacco users (smoking or smokeless or dual) at the end of the survey.

**Contact details of family member :**<br>If we are unable to reach to you at the telephone number provided by you, is it okay with you if we contact any family member suggested by you to seek the information about your tobacco usage post your hospital discharge?<br><br>Respondent consented _____1<br><br>Respondent rejected _______2<br><br>If consented - Please explain respondent all the questions that will be asked to the family member and obtain a sign/thumb impression after filling following details of the family member.
**Name of family member :**
**Contact number of family member:**
**Relation of family member with the respondent:**
**Consent: I………………………………………….. am willingly providing details of…………………………..as a contact person who can provide information on the survey questions that are explained to me by……………………. I also provide permission to consider the responses provided by ……………………as most close to my responses in terms of accuracy and use it for the purpose of the study.**<br><br>**Sign of the respondent ________**<br><br>**Sign of the investigator _______**

SECTION A: DEMOGRAPHIC INFORMATION

A1. What is the highest level of education you have completed?

	[Instruction for investigators: SELECT ONLY ONE CATEGORY] (Do not read out the options, listen to the response, confirm with cues as passed or fail and then post code accordingly.
Response Options:	ILLITERATE ☐1<br>NO FORMAL SCHOOLING BUT CAN READ ☐2<br>NO FORMAL SCHOOLING BUT CAN READ AND WRITE ☐3<br>LESS THAN PRIMARY SCHOOL COMPLETED (4th Standard completed) ☐4<br>PRIMARY SCHOOL COMPLETED (7th Standard completed) ☐5<br>LESS THAN SECONDARY SCHOOL COMPLETE (8th and 9th Standardd completed) ☐6<br>SECONDARY SCHOOL COMPLETED(10th Std completed) ☐7<br>HIGHER SECONADARYSCHOOL COMPLETED (12th Std completed) ☐8<br>COLLEGE/UNIVERSITY COMPLETED(Bachelors) ☐9<br>POST GRADUATE DEGREE COMPLETED(Masters) ☐10<br>ANY OTHER (Please specify)________________________ ☐11<br>DON'T KNOW ☐77<br>REFUSED ☐99

A2. Which of the following best describes your main work area as a source of income over the past 12 months? (single code only)

	**Government employee; Non-government employee, Self-employed; Student; Homemaker; Retired; Unemployed, able to work, or Unemployed, unable to work?**
	[INTERVIEWER: INCLUDE SUBSISTENCE FARMING AS SELF-EMPLOYED]
Response Options:	GOVERNMENT EMPLOYEE ☐1<br>NON-GOVERNMENT EMPLOYEE ☐2<br>SELF-EMPLOYED ☐3<br>STUDENT ☐4<br>HOMEMAKER ☐5<br>RETIRED ☐6<br>UNEMPLOYED, ABLE TO WORK ☐7<br>UNEMPLOYED, UNABLE TO WORK ☐8<br>DON'T KNOW ☐77<br>REFUSED ☐99

A3. Marital Status:

Response Options:	SINGLE, NEVER MARRIED ☐ 1<br>MARRIED and staying with spouse ☐ 2<br>Married but not staying with spouse ☐ 3<br>DIVORCED ☐ 4<br>SEPARATED ☐ 5<br>WIDOWED ☐ 6<br>REFUSED ☐ 99

A4. What type of telephone do you have? (Multiple codes possible)

Response Options:	ANY TELEPHONE ACCESS	Yes ☐	No ☐	→ SKIP TO A5
	LAND-LINE	Yes ☐	No ☐	
	MOBILE PHONE	Yes ☐	No ☐	
	SMART PHONE-EMAIL OR VIDEO CAPABILITY	Yes ☐	No ☐	
	DON'T KNOW	☐ 77		
	REFUSED	☐ 99		

A5. Do you send text messages? (single code only)

Response Options:	YES, I SEND TEXTS (SMS) ☐ 1<br>NO, MY PHONE CAN'T SEND TEXTS (SMS) ☐ 2

	NO, MY PHONE CAN SEND TEXTS BUT I DON'T DO IT<br>DON'T KNOW<br>REFUSED	☐ 3<br>☐ 77<br>☐ 99

A6. How often does anyone smoke tobacco inside your home? Would you say daily, weekly, monthly, less than monthly, or never?

Response Options:	DAILY<br>WEEKLY<br>MONTHLY<br>LESS THAN MONTHLY<br>NEVER<br>DON'T KNOW<br>REFUSED	☐ 1<br>☐ 2<br>☐ 3<br>☐ 4<br>☐ 5<br>☐ 77<br>☐ 99

A7. How often does anyone use smokeless tobacco inside your home? Would you say daily, weekly, monthly, less than monthly, or never?

Response Options:	DAILY<br>WEEKLY<br>MONTHLY<br>LESS THAN MONTHLY<br>NEVER<br>DON'T KNOW<br>REFUSED	☐ 1<br>☐ 2<br>☐ 3<br>☐ 4<br>☐ 5<br>☐ 77<br>☐ 99

SECTION B. SMOKELESS TOBACCO

INTRO: The next questions are about using smokeless tobacco, such as tobacco leaf, betel quid with tobacco, *sada/surti*, khaini or tobacco lime mixture, gutkha, pan masala with *tobacco, gul, gudaku, mishri*.

B1. Do you <u>currently</u> use smokeless tobacco on a daily basis, less than daily, or not at all?

Instruction:	[Present a Show card]	
Response Options:	DAILY<br>LESS THAN DAILY<br>NOT AT ALL<br>DON'T KNOW<br>REFUSED	☐ 1 → SKIP TO B4<br>☐ 2<br>☐ 3 → SKIP TO B3<br>☐ 77 → SKIP TO – SECTION C – TOBACCO SMOKING<br>☐ 99 → SKIP TO - SECTION C – TOBACCO SMOKING

B2. Have you used smokeless tobacco daily in the past

Response Options:	YES<br>NO<br>DON'T KNOW<br>REFUSED	☐ 1 → SKIP TO B8<br>☐ 2 → SKIP TO B10<br>☐ 77 → SKIP TO B10<br>☐ 99 → SKIP TO B10

B3. In the past, have you used smokeless tobacco on a daily basis, less than daily, or not at all?

Instruction:	[INTERVIEWER: IF RESPONDENT HAS DONE BOTH "DAILY" AND "LESS THAN DAILY" INTHE PAST, CHECK "DAILY" AND FOLLOW DAILY ROUTING]	
Response Options:	DAILY<br>LESS THAN DAILY<br>NOT AT ALL<br>DON'T KNOW<br>REFUSED	☐ 1 → SKIP TO B11<br>☐ 2 → SKIP TO B13<br>☐ 3 → SKIP TO SECTION C – TOBACCO SMOKING<br>☐ 77 → SKIP TO SECTION C – TOBACCO SMOKING<br>☐ 99 → SKIP TO SECTION C – TOBACCO SMOKING

[CURRENT DAILY SMOKELESS TOBACCO USERS]

B4. How old were you when you first started using smokeless tobacco daily?

Instruction:	IF DON'T KNOW OR REFUSED, ENTER "99"
Response Options:	[ ] [ ] Years old

INSTRUCTION: IF B4 = 99, ASK B5. OTHERWISE SKIP TO B6

B5. How many years ago did you first start using smokeless tobacco daily?

Instruction:	IF DON'T KNOW OR REFUSED, ENTER "99"
Response Options:	[ ] [ ] Years

B6. On an average, how many times a day do you use the following products? Also, let me know if you use the product, but not every day?

Instruction:	INTERVIEWER: IF RESPONDENT REPORTS USING THE PRODUCT LESS THAN ONCE PER WEEK, ENTER 888
Sub-Questions	READ EACH ITEM:

a. Do you chew Betel quid with tobacco every day? PER DAY
□ YES→ How many times? If YES →Record quantity and skip to b (Applies to all questions from a to i)
□ NO→a1

a1. Do you use betel quid with tobacco every week? PER WEEK
□ YES→ On average, how many times do you use betel quid with tobacco each week □ NO

b. Do you chew Khaini every day? PER DAY
□ YES→ How many times? □ NO→b1

b1. Do you use khaini every week? PER WEEK
□ YES→ On average, how many times do you use khaini each week
□ NO

c. Do you chew tobacco lime mixture every day? PER DAY
□ YES→ How many times? □ NO→c1

c1. Do you use tobacco lime mixture every week? PER WEEK
□ YES→ On average, how many times do you use tobacco lime mixture each week
□ NO

d. Do you use Gutkha daily? PER DAY
□ YES→ How many times? □ NO→d1

d1.Do you use Gutkha every week? PER WEEK
□ YES→ On average, how many times do you use Gutkha each week
□ NO

e. Do you use mava every day? PER DAY
□ YES→ How many times? □ NO→e1

e1. Do you use mava every week? PER WEEK
□ YES→ On average, how many times do you mava each week
□ NO

f. Do you use mishri every day? PER DAY
□ YES→ How many times? □ NO→f1

f1. Do you use mishri every week? PER WEEK

□ YES→ On average, how many times do you use mishri each week<br>□ NO	
g. Do you use panmasala with tobacco every day?<br>□ YES→ How many times? □ NO→g1	PER DAY
g1. Do you use panmasala with tobacco every week?<br>□ YES→ On average, how many times do you use panmasala each week<br>□ NO	PER WEEK
h. Do you use Nasal use of snuff, every day?<br>□ YES→ How many times? □ NO→h1	PER DAY
h1. Do you use nasal use of snuff every week?<br>□ YES→ On average, how many times do you use snuff each week?<br>□ NO	PER WEEK
i. Do you use any other tobacco product [FILL PRODUCT] every day? (Specify type)<br>□ YES→How many per day?<br>□ NO	PER DAY
i1. Do you use any tobacco [FILL PRODUCT} every week?<br>□ YES→On average, how many times do you use [FILL PRODUCT] each week?<br>□ NO	PER WEEK

B7. Now I will ask few more questions about your smokeless tobacco use behavior

Instructions	Read out all options	
Sub questions:	1. How soon after you wake up do you use your first tobacco product?	Within 5 min (3)<br>6–30 min (2)<br>31–60 min (1)<br>After 60 min (0)
	2. How often do you intentionally swallow tobacco juice?	Always (2)<br>Sometimes (1)<br>Never (0)
	3. Which chew would you hate to give up most?	The first one in the morning (1)<br>Any other (0)
	4. How many cans/pouches (packets of tobacco product) per week do you use?	More than 3 (2)<br>2–3 (1)<br>1 (0)
	5. Do you chew more frequently during the first hours after awakening than during the rest of the day?	Yes (1)<br>No (0)
	6. Do you chew if you are so ill that you are in bed most of the day?	Yes (1)<br>No (0)

INSTRUCTION: SKIP TO B15

[CURRENT LESS THAN DAILY SMOKELESS TOBACCO USERS]

B8. How old were you when you first started using smokeless tobacco daily?

Instruction:	IF DON'T KNOW OR REFUSED, ENTER "99"
Response Options:	__ __ Years **old**

INSTRUCTION: IF B8 = 99, ASK B9. OTHERWISE SKIP TO B10.	
**B9. How many years ago did you first start using smokeless tobacco daily?**	
Instruction:	IF DON'T KNOW OR REFUSED, ENTER "99"
Response Options:	[ ][ ] Years
**B10. How many times a week do you usually use the following?**	
Instruction 1:	INTERVIEWER: IF RESPONDENT REPORTS DOING THE ACTIVITY WITHIN THE PAST 30 DAYS, BUT LESS THAN ONCE PER WEEK, RECORD 888

	Product	Quantity
Sub-Question	a. Betel quid with tobacco	PER WEEK
	b. Khaini	PER WEEK
	c. tobacco lime mixture	PER WEEK
	d. Gutkha	PER WEEK
	e. Mava	PER WEEK
	f. Mishri	PER WEEK
	g. Paan masala with tobacco	PER WEEK
	h. Nasal use of snuff	PER WEEK
	i. Any others? Specify type: ________________	PER WEEK

INSTRUCTION: SKIP TO B15

[FORMER SMOKELESS TOBACCO USERS]	
**B11. How old were you when you first started using smokeless tobacco daily?**	
Instruction:	IF DON'T KNOW OR REFUSED,ENTER "99"
Response Options:	[ ][ ] Years old
**INT: IF B11 = 99, ASK B12. OTHERWISE SKIP TO B13.**	
**B12. How many years ago did you first start using smokeless tobacco daily?**	
Instruction:	IF DON'T KNOW OR REFUSED,ENTER "99"
Response Options:	[ ][ ] Years
**B13. How long has it been since you stopped using smokeless tobacco?**	
Instruction:	INTERVIEWER: ONLY INTERESTED IN WHEN RESPONDENT STOPPED USING SMOKELESS TOBACCO REGULARLY -- DO NOT INCLUDE RARE INSTANCES OF USING SMOKELESS TOBACCO<br>CHECK UNIT AND RECORD NUMBER
Response Options:	No. of units: ________________

	Units: (Select from the options below)<br>YEARS ☐ 1<br>MONTHS ☐ 2<br>DAYS ☐ 3<br>LESS THAN ONE DAY ☐ 4<br>DON'T KNOW ☐ 77<br>REFUSED ☐ 99

B14. What was the reason for your quitting smokeless tobacco?

Instruction:	PLEASE PROMPT EACH OPTION AND RECORD YES/NO FOR EACH
Response Options:	1. Current health problem Yes ☐1 No ☐2 DON'T KNOW ☐ 7 REFUSED ☐ 9<br>2. Concern for future health Yes ☐1 No ☐2 DON'T KNOW ☐ 7 REFUSED ☐ 9<br>3. Pressure from family Yes ☐1 No ☐2 DON'T KNOW ☐ 7 REFUSED ☐ 9<br>4. Pressure from friends Yes ☐1 No ☐2 DON'T KNOW ☐ 7 REFUSED ☐ 9<br>5. Advice from your doctor Yes ☐1 No ☐2 DON'T KNOW ☐ 7 REFUSED ☐ 9<br>6. Illness or death of a friend or relative Yes ☐1 No ☐2 DON'T KNOW ☐ 7 REFUSED ☐ 9<br>7. Personal appearance (Bad breath, stains teeth etc.) Yes ☐1 No ☐2 DON'T KNOW ☐ 7 REFUSED ☐ 9<br>8. Financial reason Yes ☐1 No ☐2 DON'T KNOW ☐ 7 REFUSED ☐ 9<br>9. My smoking causing harm to family members Yes ☐1 No ☐2 DON'T KNOW ☐ 7 REFUSED ☐ 9<br>Among the above, which is the one most important reason according to you?<br>☐1 ☐2 ☐3 ☐4 ☐5 ☐6 ☐7 ☐8 ☐9

INSTRUCTION: SKIP TO SECTION C – TOBACCO SMOKING

B15. Have you visited a doctor or a health care provider in the 12 months before you were admitted to this hospital for any reason of personal health?

Response Options:	YES<br>NO<br>REFUSED	☐ 1<br>☐ 2 → SKIP TO Section C Tobacco smoking<br>☐ 9 → SKIP TO Section C Tobacco smoking

B16. During any visit to a doctor or health care provider in the past 12 months, were you asked if you use smokeless tobacco?

Response Options:	YES<br>NO<br>REFUSED	☐ 1<br>☐ 2 → SKIP TO Section C Tobacco smoking<br>☐ 9 → SKIP TO Section C Tobacco smoking

B17. During any visit to a doctor or health care provider in the past 12 months, were you advised to quit smokeless tobacco use?

Response Options:	YES<br>NO<br>REFUSED	☐ 1<br>☐ 2<br>☐ 9

SECTION C. TOBACCO SMOKING

INTRO: I would now like to ask you some questions about <u>smoking</u> tobacco, including bidis, cigarettes, hookah. Please do not answer about smokeless tobacco at this time.

C1. Do you <u>currently</u> smoke tobacco on a daily basis, less than daily, or not at all?

Instruction:	[Present a Show card]	
Response	DAILY	☐1 → SKIP TO C4

Options:	LESS THAN DAILY<br>NOT AT ALL<br>DON'T KNOW<br>REFUSED................	☐2<br>☐3 → SKIP TO C3<br>☐7 → SKIP TO SECTION E - KNOWLEDGE<br>☐9 → SKIP TO SECTION E - KNOWLEDGE

C2. Have you smoked tobacco daily in the past?

Response Options:	YES<br>NO<br>DON'T KNOW<br>REFUSED................	☐1 → SKIP TO C8<br>☐2 → SKIP TO C10<br>☐7 → SKIP TO C10<br>☐9 → SKIP TO C10

C3. In the past, have you smoked tobacco on a daily basis, less than daily, or not at all?

Instruction:	[INTERVIEWER: IF RESPONDENT HAS DONE BOTH "DAILY" AND "LESS THAN DAILY" INTHE PAST, CHECK "DAILY" AND FOLLOW DAILY ROUTING]	
Response Options:	DAILY<br>LESS THAN DAILY<br>NOT AT ALL<br>DON'T KNOW<br>REFUSED................	☐1 → SKIP TO C11<br>☐2 → SKIP TO C13<br>☐3 → SKIP TO SECTION E - KNOWLEDGE<br>☐7 → SKIP TO SECTION E – KNOWLEDGE<br>☐9 → SKIP TO SECTION E - KNOWLEDGE

[CURRENT DAILY SMOKERS]

C4. How old were you when you first started smoking tobacco daily?

Instruction:	IF DON'T KNOW OR REFUSED,ENTER "99"
Response Options:	☐ ☐ Years old

INSTRUCTION: IF C4 = 99, ASK C5. OTHERWISE SKIP TO C6.

C5. How many years ago did you first start smoking tobacco daily?

Instruction:	IF DON'T KNOW OR REFUSED, ENTER "99"
Response Options:	☐ ☐ Years

C6. On average, how many of the following products do you currently smoke each day? Also, let me know if you smoke the product, but not every day.

Instruction:	INTERVIEWER: IF RESPONDENT REPORTS SMOKING THE PRODUCT LESS THAN ONCE PER WEEK, ENTER 888 UNDER PART X1.<br>IF RESPONDENT REPORTS IN PACKS OR CARTONS, PROBE TO FIND OUT HOW MANY ARE IN EACH AND CALCULATE TOTAL NUMBER
Sub-Questions	READ EACH ITEM:

a. Do you smoke cigarettes every day?<br>☐ YES→ How many? If YES →Record quantity and skip to b (Applies to all questions from a to d)<br>☐ NO→a1	PER DAY
a1. [IF B6a=888] Do you smoke cigarettes every week?<br>☐ YES→On average, how many cigarettes do you currently smoke each week? ☐ NO	PER WEEK
b. Do you smoke Bidis every day?<br>☐ YES→ How many? ☐ NO→b1	PER DAY
b1. [IF B6b=888] Do you smoke bidis every week?<br>☐ YES→On average, how many bidis do you currently smoke each week? ☐ NO	PER WEEK
c. Do you smoke hukkah every day?	PER DAY

□ YES→ How many hukkah sessions per day? □ NO→c1
c1. IF B6c=888] DO you smoke hukkah every week? PER WEEK<br>□ YES→On average, how many hukkah sessions do you currently participate in each week? □ NO
d. Do you smoke any other products every day? PER DAY<br>□ YES→How many per day? □ NO →d1<br>(Specify type)
d1. Do you smoke any other products every day? PER WEEK<br>□ YES→How many per day? □ NO<br>(Specify type)

C7. Now I will ask few more questions about your smoked tobacco use behavior?

Response Options:	1. How soon after you wake up do you smoke your first cigarette/bidi/hookah?	Within 5 minutes (3)<br>6-30 minutes (2)<br>31-60 minutes (1)<br>After 60 minutes (0)
	2. Do you find it difficult to refrain from smoking in places where it is forbidden?	Yes (1)<br>No (0)
	3. Which cigarette/bidi/hookah would you hate most to give up?	The first in the morning (1)<br>Any other (0)
	4. How many cigarettes/bidis/hookah sessions per day do you smoke?	31 or more (3)<br>21-30 (2)<br>11-20 (1)<br>10 or less (0)
	5. Do you smoke more frequently during the first hours after awakening than during the rest of the day?	Yes (1)<br>No (0)
	6. Do you smoke even if you are so ill that you are in bed most of the day?	Yes (1)<br>No (0)

SKIP TO C 15

[CURRENT LESS THAN DAILY SMOKERS]

C8. How old were you when you first started smoking tobacco daily?

Instruction:	IF DON'T KNOW OR REFUSED, ENTER "99"
Response Options:	☐☐ Years old

INSTRUCTION: IF C8 = 99, ASK C9. OTHERWISE SKIP TO C10.

C9. How many years ago did you first start smoking tobacco daily?

Instruction:	IF DON'T KNOW OR REFUSED, ENTER "99"
Response Options:	☐☐ Years

C10. How many of the following do you currently smoke during a usual week?

Instruction 1:	INTERVIEWER: IF RESPONDENT REPORTS DOING THE ACTIVITY WITHIN THE PAST 30 DAYS, BUT LESS THAN ONCE PER WEEK, RECORD 888

Instruction 2:	INTERVIEWER: IF RESPONDENT REPORTS IN PACKS OR CARTONS, PROBE TO FIND OUT HOW MANY ARE IN EACH AND CALCULATE TOTAL NUMBER<br>READ EACH ITEM:	
Sub-Question		
	Product	Quantity
	j. Cigarettes?	PER WEEK
	k. Bidis?	PER WEEK
	l. Number of hukkah sessions per week?	PER WEEK
	m. Any others?<br>Specify type: ________________	PER WEEK

SKIP TO C 15

[FORMER SMOKERS]

C11. How old were you when you first started smoking tobacco daily?

Instruction:	IF DON'T KNOW OR REFUSED, ENTER "99"
Response Options:	[ ] [ ] Years old

INT: IF C11 = 99, ASK C12. OTHERWISE SKIP TO C13.

C12. How many years ago did you first start smoking tobacco daily?

Instruction:	IF DON'T KNOW OR REFUSED,ENTER "99"
Response Options:	[ ] [ ] Years

C13. How long has it been since you stopped smoking?

Instruction:	INTERVIEWER: ONLY INTERESTED IN WHEN RESPONDENT STOPPED SMOKING REGULARLY -- DO NOT INCLUDE RARE INSTANCES OF SMOKING CHECK UNIT AND RECORD NUMBER
Response Options:	No. of units: ________________<br><br>Units: (Select from the options below)<br>YEARS ☐1<br>MONTHS ☐2<br>WEEKS ☐3<br>DAYS ☐4<br>LESS THAN ONE DAY ☐5<br>DON'T KNOW ☐7<br>REFUSED ☐9

C14. What was the reason for your quitting smoking tobacco?

Instruction:	PLEASE PROMPT EACH OPTION AND RECORD YES/NO FOR EACH
Response Options:	1. Current health problem Yes ☐1 No ☐2 DON'T KNOW ☐ 7 REFUSED ☐ 9<br>2. Concern for future health Yes ☐1 No ☐2 DON'T KNOW ☐ 7 REFUSED ☐ 9<br>3. Pressure from family Yes ☐1 No ☐2 DON'T KNOW ☐ 7 REFUSED ☐ 9<br>4. Pressure from friends Yes ☐1 No ☐2 DON'T KNOW ☐ 7 REFUSED ☐ 9<br>5. Advice from your doctor Yes ☐1 No ☐2 DON'T KNOW ☐ 7 REFUSED ☐ 9<br>6. Illness or death of a friend or relative Yes ☐1 No ☐2 DON'T KNOW ☐ 7 REFUSED ☐ 9

	7. Personal appearance (Bad breath, stains teeth etc.) Yes ☐1 No ☐2 DON'T KNOW ☐ 7 REFUSED ☐ 9<br>8. Financial reason Yes ☐1 No ☐2 DON'T KNOW ☐ 7 REFUSED ☐ 9<br>9. My smoking causing harm to family members Yes ☐1 No ☐2 DON'T KNOW ☐ 7 REFUSED ☐ 9<br><br>Among the above, which is the one most important reason according to you?<br>☐1 ☐2 ☐3 ☐4 ☐5 ☐6 ☐7 ☐8

INSTRUCTION: SKIP TO SECTION E - KNOWLEDGE

C15. Have you visited a doctor or a health care provider in the 12 months before you were admitted to this hospital for any reason of personal health?

Response Options:	YES	☐ 1
	NO	☐ 2 → SKIP TO SECTION D
	REFUSED	☐ 9 → SKIP TO SECTION D

C16. During any visit to a doctor or health care provider in the past 12 months, were you asked if you use smoked tobacco?

Response Options:	YES	☐ 1
	NO	☐ 2 → SKIP TO SECTION D
	REFUSED	☐ 9 → SKIP TO SECTION D

C17. During any visit to a doctor or health care provider in the past 12 months, were you advised to quit smoked tobacco use?

Response Options:	YES	☐ 1
	NO	☐ 2
	REFUSED	☐ 9

SECTION : D DETAILS ON QUITTING (ONLY FOR CURRENT DAILY AND LESS THAN DAILY SMOKING AND SMOKELESS TOBACCO USERS)

D1. Before this interview, has anyone in the hospital asked whether you use tobacco?

Response Options:	YES	☐ 1
	NO	☐ 2
	REFUSED	☐ 9

D2. Before this interview, has anyone in the hospital advised you to quit tobacco use?

Response Options:	YES	☐ 1
	NO	☐ 2
	REFUSED	☐ 9

D3. Have you ever stopped using tobacco for ONE DAY or longer because you were trying to quit tobacco?

	YES	☐ 1
	NO	☐ 2
	REFUSED	☐ 9

D4. During the PAST 12 MONTHS, have you stopped using tobacco for ONE DAY or longer because you were trying to quit tobacco?

Response Options	YES	☐ 1
	NO	☐ 2 SKIP TO D 7
	DON'T KNOW	☐ 77 SKIP TO D 7

	REFUSED ☐ 99 SKIP TO D 7

D5. What was the concern / reason when you tried to quit tobacco in the last 12 months?

Instruction:	PLEASE PROMPT EACH OPTION AND RECORD YES/NO FOR EACH
Response Options:	1. Current health problem Yes ☐1 No ☐2 DON'T KNOW ☐ 7 REFUSED ☐ 9<br>2. Concern for future health Yes ☐1 No ☐2 DON'T KNOW ☐ 7 REFUSED ☐ 9<br>3. Pressure from family Yes ☐1 No ☐2 DON'T KNOW ☐ 7 REFUSED ☐ 9<br>4. Pressure from friends Yes ☐1 No ☐2 DON'T KNOW ☐ 7 REFUSED ☐ 9<br>5. Advice from your doctor Yes ☐1 No ☐2 DON'T KNOW ☐ 7 REFUSED ☐ 9<br>6. Illness or death of a friend or relative Yes ☐1 No ☐2 DON'T KNOW ☐ 7 REFUSED ☐ 9<br>7. Personal appearance (Bad breath, stains teeth etc.) Yes ☐1 No ☐2 DON'T KNOW ☐ 7 REFUSED ☐ 9<br>8. Financial reason Yes ☐1 No ☐2 DON'T KNOW ☐ 7 REFUSED ☐ 9<br>9. My smoking causing harm to family members Yes ☐1 No ☐2 DON'T KNOW ☐ 7 REFUSED ☐ 9<br><br>Among the above, which is the one most important reason according to you?<br>☐1 ☐2 ☐3 ☐4 ☐5 ☐6 ☐7 ☐8

D6. During the past 12 months, did you use any of the following to try to stop using tobacco?

Sub-Questions:	INSTRUCTIONS: READ EACH ITEM	
	a. Counseling, including at a tobacco cessation clinic?	YES NO REFUSED
	b. Nicotine replacement therapy, such as the patch or gum?	YES NO REFUSED
	c. Other prescription medications, for example Bupropion/ Champix?	YES NO REFUSED
	d. Traditional medicines, for example Ayurvedic, Homeopathic, Unani?	YES NO REFUSED
	e. A quit line or a smoking cessation telephone support line?	YES NO REFUSED
	f. Switching to smokeless tobacco?	YES NO REFUSED
	g. Quit on your own?	YES NO REFUSED
	h. Anything else? Specify:______	YES NO REFUSED

D7. What is your plan about your tobacco use after you leave the hospital?

Response Options:	I WILL STAY QUIT AFTER I LEAVE ☐ 1<br>I WILL TRY TO QUIT AFTER I LEAVE ☐ 2<br>I DON'T PLAN TO QUIT ☐ 3 ASK D10<br>DON'T KNOW ☐ 7 ASK D10<br>REFUSED ☐ 9

D8. Are you seriously thinking about quitting tobacco use within?

Response Options	NEXT 30 days ☐ 1<br>Not in next 30 days but in NEXT 6 months ☐ 2<br>Not in the next 6 months ☐ 3<br>Already quit ☐ 4<br>DON'T KNOW ☐ 7<br>REFUSED ☐ 9

INSTRUCTIONS: SKIP TO NEXT SECTION

D9. If you were going to quit tobacco use, how likely would you be to ask a DOCTOR for advice?

Response Options	Very likely	☐ 1
	Somewhat likely	☐ 2
	Not likely at all	☐ 3
	Don't know	☐ 7
	Refused	☐ 9

D10. During the past 12 months, have you felt pressure from other people to quit your tobacco use?

Response Options	YES	☐ 1 ASK D11
	NO	☐ 2 SKIP TO NEXT SECTION
	DON'T KNOW	☐ 77 SKIP TO NEXT SECTION
	REFUSED	☐ 99 SKIP TO NEXT SECTION

D11. If Yes, from whom?

FROM FAMILY MEMBERS	Yes ☐1	No ☐2	DON'T KNOW ☐ 7	REFUSED ☐ 9
FROM FRIENDS	Yes ☐1	No ☐2	DON'T KNOW ☐ 7	REFUSED ☐ 9
FROM YOUR PHYSICIAN	Yes ☐1	No ☐2	DON'T KNOW ☐ 7	REFUSED ☐ 9
YOUR CO-WORKERS	Yes ☐1	No ☐2	DON'T KNOW ☐ 7	REFUSED ☐ 9
ANY OTHERS ________				

SECTION E: KNOWLEDGE (FOR ALL PATIENTS)

E1. Based on what you know or believe, does smoking tobacco cause serious illness?

Response Options:	YES, SMOKING CAUSES SERIOUS ILLNESS	☐ 1
	NO, SMOKING DOES NOT CAUSE SERIOUS ILLNESS	☐ 2
	DON'T KNOW	☐ 7
	REFUSED	☐ 9

E2. Based on what you know or believe, does smokeless tobacco cause serious illness?

Response Options:	YES, SMOKELESS TOBACCO CAUSES SERIOUS ILLNESS	☐ 1
	NO, SMOKELESS TOBACCO DOES NOT CAUSE SERIOUS ILLNESS	☐ 2
	DON'T KNOW	☐ 7
	REFUSED	☐ 9

E3. Based on what you know or believe, does smoking cause the following?

Response Options:	HEART ATTACK	YES ☐ 1	NO ☐ 2	DK ☐ 7
	CANCER	YES ☐ 1	NO ☐ 2	DK ☐ 7
	STROKE	YES ☐ 1	NO ☐ 2	DK ☐ 7

E4. Based on what you know or believe, does smokeless tobacco cause cancer?

Response Options:	YES, SMOKELESS TOBACCO CAUSES CANCER	☐ 1
	NO, SMOKELESS TOBACCO DOES NOT CAUSE CANCER	☐ 2
	DON'T KNOW	☐ 7
	REFUSED	☐ 9

FOR PATIENTS WHO ARE NOT CURRENT TOBACCO USERS SKIP TO SECTION G : PATIENT HEALTH QUESTIONNAIRE

SECTION F: PERCEPTIONS FOR current tobacco users only

F1. Now I want to ask about your opinions. Do think tobacco use has harmed your health?

Response Options:	NOT AT ALL	☐ 1
	A LITTLE BIT	☐ 2
	SOME	☐ 3

	A LOT ☐ 4<br>DON'T KNOW ☐ 7

F2. In your opinion, is tobacco use a cause of the illness you are in the hospital for?

Response Options:	NOT AT ALL ☐ 1<br>A LITTLE BIT ☐ 2<br>SOME ☐ 3<br>A LOT ☐ 4<br>DON'T KNOW ☐ 7

F3. Do you think quitting tobacco use now would improve your health?

Response Options	NOT AT ALL ☐ 1<br>A LITTLE BIT ☐ 2<br>SOME ☐ 3<br>A LOT ☐ 4<br>DON'T KNOW ☐ 7

F4. How important is it to you to quit tobacco after leaving the hospital?

Response Options	NOT IMPORTANT ☐ 1<br>SOMEWHAT IMPORTANT ☐ 2<br>VERY IMPORTANT ☐ 3<br>DON'T KNOW ☐ 7<br>REFUSED ☐ 9

F5. How confident are you that you could stop tobacco after leaving the hospital, if you decided to?

Response Options	NOT CONFIDENT ☐ 1<br>SOMEWHAT CONFIDENT ☐ 2<br>VERY CONFIDENT ☐ 3<br>DON'T KNOW ☐ 7<br>REFUSED ☐ 9

F6. How motivated are you to quit tobacco after leaving the hospital??

Response Options	NOT MOTIVATED ☐ 1<br>SOMEWHAT MOTIVATED ☐ 2<br>VERY MOTIVATED ☐ 3<br>DON'T KNOW ☐ 7<br>REFUSED ☐ 9

SECTION G: PATIENT HEALTH QUESTIONNAIRE FOR ALL PATIENTS

Over the last 2 weeks, how often have you been bothered by the following problems?	Not at all	Several days	More than half the days	Nearly every day
Feeling nervous, anxious, or on edge	1	2	3	4
Not being able to stop or control worrying	1	2	3	4
Feeling down, depressed, or hopeless	1	2	3	4
Little interest or pleasure in doing things	1	2	3	4

Those are all of the questions I have. Thank you very much for participating in this important survey.

FOLLOW-UP SURVEY

**"Are you (patient's name)?"**		
Response Options:	YES<br>NO	☐1<br>☐2 → SKIP TO A8

SECTION A. SMOKELESS TOBACCO

I would like to ask you some questions about using smokeless tobacco, such as tobacco leaf, betel quid with tobacco, sada/surti, khaini or tobacco lime mixture, gutkha, pan masala with zarda, gul, gudaku, mishri.

A1. Since leaving the hospital, have you used any smokeless tobacco?

Response	YES<br>NO<br>DON'T KNOW<br>REFUSED……………..	☐1 →SKIP TO A3<br>☐2 → CONGRATULATE AND SKIP TO A2<br>☐7 → SKIP TO SECTION B: SMOKED TOBACCO<br>☐9 → SKIP TO SECTION B: SMOKED TOBACCO

A2. What was the concern / reason for you to not to use tobacco after discharge from the hospital?

Response Options:

1. Current health problem Yes ☐1 No ☐2 DON'T KNOW ☐ 7 REFUSED ☐ 9
2. Concern for future health Yes ☐1 No ☐2 DON'T KNOW ☐ 7 REFUSED ☐ 9
3. Pressure from family Yes ☐1 No ☐2 DON'T KNOW ☐ 7 REFUSED ☐ 9
4. Pressure from friends Yes ☐1 No ☐2 DON'T KNOW ☐ 7 REFUSED ☐ 9
5. Advice from your doctor Yes ☐1 No ☐2 DON'T KNOW ☐ 7 REFUSED ☐ 9
6. Illness or death of a friend or relative
   Yes ☐1 No ☐2 DON'T KNOW ☐ 7 REFUSED ☐ 9
7. Personal appearance (Bad breath, stains teeth etc.)
   Yes ☐1 No ☐2 DON'T KNOW ☐ 7 REFUSED ☐ 9
8. Financial reason Yes ☐1 No ☐2 DON'T KNOW ☐ 7 REFUSED ☐ 9
9. My smoking causing harm to family members
   Yes ☐1 No ☐2 DON'T KNOW ☐ 7 REFUSED ☐ 9

Among the above, which is the one most important reason according to you?
☐1 ☐2 ☐3 ☐4 ☐5 ☐6 ☐7 ☐8 ☐ 9

INSTRUCTIONS: SKIP TO A7

A3. How many days after you left the hospital did you first use smokeless tobacco?

Response Options:			**DAYS**
	**Enter 77 if DON'T KNOW or 99 if REFUSED**		

A4. Did you use any smokeless tobacco, even a chew, during your stay in the hospital?

Response Options:	YES<br>NO<br>DON'T KNOW<br>REFUSED	☐ 1<br>☐ 2<br>☐ 7<br>☐ 9

A5. When you were in the hospital, did anyone in the hospital advise you to quit using smokeless tobacco?

	YES<br>NO<br>DON'T KNOW<br>REFUSED	☐ 1<br>☐ 2<br>☐ 7<br>☐ 9

A6. After getting discharged from the hospital did you try to stop using smokeless tobacco?

	YES<br>NO<br>DON'T KNOW<br>REFUSED	☐ 1 ASK A7<br>☐ 2 SKIP TO SECTION B: SMOKED TOBACCO<br>☐ 7 SKIP TO SECTION B: SMOKED TOBACCO<br>☐ 9 SKIP TO SECTION B: SMOKED TOBACCO

**A7. Since leaving the hospital, did you use any of the following to try to stop using smokeless tobacco?**	
Sub-Question	a. Counseling, including at a tobacco cessation clinic
	b. Nicotine replacement therapy, such as the patch or gum
	c. Other prescription medications, for example Bupropion/Champix
	d. Traditional medicines, for example Ayurvedic, Homeopathic, Unani
	e. A quit line or a smoking telephone support line
	f. Switching to non-tobacco chewing product
	g. Quit on your own
	h. Anything else? Please specify________
**SKIP TO SECTION B**	
**[IF USING PROXY]**	
**A8. Since leaving the hospital, has (Name of the patient) used any smokeless tobacco product?**	
Response Options:	YES ☐1<br>NO ☐2 → SKIP TO END<br>DON'T KNOW ☐7 → SKIP TO END<br>REFUSED................ ☐9 → SKIP TO END
**A9. How many days after leaving the hospital he first used any smokeless product?**	
Response Options:	☐ ☐ **DAYS**
Instructions	**Enter 77 if DON'T KNOW or 99 if REFUSED**
**SKIP TO B8**	
**SECTION B: SMOKING TOBACCO**	
I would now like to ask you some questions about smoking tobacco, including bidis, cigarettes, hookah. Please do not answer about smokeless tobacco at this time.	
**B1. Since leaving the hospital, have you smoked any tobacco product even once?**	
Response Options:	YES ☐1 → SKIP TO B3<br>NO ☐2 → CONGRATULATE AND SKIP TO B2<br>DON'T KNOW ☐7 → SKIP TO B1<br>REFUSED................ ☐9 → SKIP TO B1
**B2. What was the reason for you to not to smoke after discharge from the hospital?**	

1. Current health problem Yes ☐1 No ☐2 DON'T KNOW ☐ 7 REFUSED ☐ 9
2. Concern for future health Yes ☐1 No ☐2 DON'T KNOW ☐ 7 REFUSED ☐ 9
3. Pressure from family Yes ☐1 No ☐2 DON'T KNOW ☐ 7 REFUSED ☐ 9
4. Pressure from friends Yes ☐1 No ☐2 DON'T KNOW ☐ 7 REFUSED ☐ 9
5. Advice from your doctor Yes ☐1 No ☐2 DON'T KNOW ☐ 7 REFUSED ☐ 9
6. Illness or death of a friend or relative
   Yes ☐1 No ☐2 DON'T KNOW ☐ 7 REFUSED ☐ 9
7. Personal appearance (Bad breath, stains teeth etc.)

	Yes ☐1 No ☐2 DON'T KNOW ☐ 7 REFUSED ☐ 9<br>8. Financial reason Yes ☐1 No ☐2 DON'T KNOW ☐ 7 REFUSED ☐ 9<br>9. My smoking causing harm to family members<br>Yes ☐1 No ☐2 DON'T KNOW ☐ 7 REFUSED ☐ 9<br><br>Among the above, which is the one most important reason according to you?<br>☐1 ☐2 ☐3 ☐4 ☐5 ☐6 ☐7 ☐8

INSTRUCTIONS: SKIP TO NEXT SECTION – Q. B7

B3. How many days after you left the hospital did you first smoke?

Response Options:	__ __ **DAYS**
Instruction	**Enter 77 if DON'T KNOW or 99 if REFUSED**

B4. Did you smoke any tobacco, even a puff, during your stay in the hospital

Response Options:	YES ☐ 1<br>NO ☐ 2<br>DON'T KNOW ☐ 7<br>REFUSED ☐ 9

B5. When you were in the hospital, did anyone in the hospital advise you to quit smoking tobacco?

Response Options:	YES ☐ 1<br>NO ☐ 2<br>DON'T KNOW ☐ 7<br>REFUSED ☐ 9

B6. After getting discharged from the hospital did you try to stop smoking tobacco?

Response Options:	YES ☐ 1<br>NO ☐ 2 SKIP TO NEXT SECTION<br>DON'T KNOW ☐ 7 SKIP TO NEXT SECTION<br>REFUSED ☐ 9 SKIP TO NEXT SECTION

B7. Since leaving the hospital, did you use any of the following to try to stop smoking tobacco?

Sub-Question	
	a. Counseling, including at a tobacco cessation clinic? ☐
	b. Nicotine replacement therapy, such as the patch or gum? ☐
	c. Other prescription medications, for example Bupropion/Champix? ☐
	d. Traditional medicines, for example Ayurvedic, Homeopathic, Unani? ☐
	e. A quit line or a smoking telephone support line? ☐
	f. Switching to smokeless tobacco? ☐
	g. Quit on your own? ☐
	h. Anything else? Specify:______

[IF USING PROXY]

**B8. Since leaving the hospital, has (Name of the patient) used any smoking tobacco product?**		
Response Description:	YES<br>NO<br>DON'T KNOW<br>REFUSED……………..	☐1<br>☐2 → SKIP TO B3<br>☐7 → SKIP TO B3<br>☐9 → SKIP TO B3

**B9. How many days after leaving the hospital he/she first used any smoking tobacco product?**			
Sub-Question			**DAYS**
	**Enter 77 if DON'T KNOW or 99 if REFUSED**		
