# Supplementary material for: Post-discharge tobacco abstinence in a Mumbai hospital after implementation of tobacco cessation counseling: A pragmatic evaluation of the LifeFirst program
Source: PLoS One. 2024 Nov 12;19(11):e0312319. doi: 10.1371/journal.pone.0312319 (PMC11556754; doi:10.1371/journal.pone.0312319)
Supplement: S1 Table — (DOCX) [file pone.0312319.s002.docx]

**S1 Table.** LifeFirst protocol and contents by session

LifeFirst tobacco dependence treatment programme is implemented in various settings like healthcare facilities including primary and tertiary care centers; at outpatient and inpatient levels, workplaces and schools. LifeFirst counselling for adult tobacco users in healthcare settings involves behavioral modification counselling based on the Transtheoretical Model of health behavior change. While the Counselling sessions focus on the skills needed in counselling it also adopts several techniques of motivational interviewing.

Following is a brief description of the LifeFirst protocol being followed at Prince Aly Khan Hospital where the intervention was implemented for the admitted patients (inpatients).

| Session # | Schedule | Details |
| --- | --- | --- |
| First Session | Day 0 | First session involves recording personal history, medical history of the client, understanding the knowledge of the patient about harmful effects of tobacco, understanding pattern of tobacco usage by the client, diagnosing the type of addiction of the patient based on the extent of dependence as ascertained by the FTND scores of the client and the stage of change they are currently in.  While the relevant information about above parameters is recorded/documented – an attempt is also made by the counsellor to understand the underlying motivating factors for the client by exploring about health, social and economic factors influencing tobacco use for the client.  Depending upon their stage of readiness based on the Transtheoretical Model of Change (TTM), the counseling conversation is tailored. If the person is not ready, then the focus is on arriving at the probable motivation that is derived from the conversation with the client, by asking the appropriate questions. The client can also be made to visualize their life without tobacco and discover what could be the good and the bad part of living without tobacco. This information can then be used to weave in the counseling conversation. During this stage, the emphasis is also on providing psychoeducation about tobacco.  If the client becomes ready to quit, then he is assisted by designing a plan collaboratively and with empathy, believing that the client knows what is doable for them. The plan may comprise of scheduling a quit date, identifying their triggers and also devising appropriate alternative ways to cope with them. During this time the counselor may also help the client build certain life skills that can help them cope with stress, build refusal skills, enhance their emotional tolerance and also provide them with certain evidence-based strategies that can help them to calm down their physiological urges. |
| Follow-up 1 | 7 days | Counsellors assess the potential difficulty that the patient might face during his/her quit journey by gauging the motivation vis-a vis the reasons for making quit attempt. After understanding any challenges that the patient might have experienced – the status of tobacco use for the client is inquired and recorded using 7 days point prevalence abstinence (7 days PPA) and continued abstinence from the date of discharge. Here the focus is to sustain the motivation of the client, by gently reminding them of their initial reason for quitting and also reinforcing them for their attempts made towards it. The strategies used by them to abstain can then be stretched to be applied at other times too. |
| Follow-up 2 | 15 days | At second follow-up the attempt is done along the same lines, to understand the challenges faced by the patient during quit journey and tobacco use status is recorded along the same lines. In any of the attempts if the client slips or relapses, then the reason for that deviation is used to further strengthen their skills in abstaining. |
| Follow-up 3 | 1 month | More or less a similar pattern is followed by the counsellor at every follow-up. The idea at every follow-up is to further work on sustaining the motivation of the patient to quit using motivational interviewing technique and address the challenges faced by respective patient. The plan can be readjusted or modified to meet the challenges of the client. |
| Follow-up 4 | 2 months |  |
| Follow-up 5 | 4 months |  |
| Follow-up 6 | 6 months | At sixth follow-up the final outcome for the case is recorded. At every stage reminding and acknowledging the client for their successful attempts to move forwarded in the quitting process and abstinence is very essential. |
