# Supplementary material for: Post-discharge tobacco abstinence in a Mumbai hospital after implementation of tobacco cessation counseling: A pragmatic evaluation of the LifeFirst program
Source: PLoS One. 2024 Nov 12;19(11):e0312319. doi: 10.1371/journal.pone.0312319 (PMC11556754; doi:10.1371/journal.pone.0312319)
Supplement: S2 Table — (DOCX) [file pone.0312319.s003.docx]

**S2 Table.** Tobacco product use in Pre- and Post-implementation samples

|  | Pre-implementation of LifeFirst | | Post-implementation of LifeFirst | |
| --- | --- | --- | --- | --- |
|  | N | % | N | % |
| Current smoked product use |  |  |  |  |
| All | 185 | 100.0 | 200 | 100.0 |
| Manufactured cigarettes |  |  |  |  |
| Daily/Weekly | 151 | 81.6 | 167 | 83.5 |
| No use | 34 | 18.4 | 33 | 16.5 |
| Bidis |  |  |  |  |
| Daily/Weekly | 54 | 29.2 | 57 | 28.5 |
| No use | 131 | 70.8 | 143 | 71.5 |
| Hukkah |  |  |  |  |
| Daily/Weekly | 5 | 2.7 | 6 | 3.0 |
| No use | 180 | 97.3 | 194 | 97.0 |
| Other smoked products |  |  |  |  |
| Daily/Weekly | -- | -- | 2 | 1.0 |
| No use | 185 | 100.0 | 198 | 99.0 |
| Current smokeless product use |  |  |  |  |
| All | 290 | 100.0 | 409 | 100.0 |
| Betel quid |  |  |  |  |
| Daily/Weekly | 96 | 33.1 | 104 | 25.4 |
| No use | 194 | 66.9 | 305 | 74.6 |
| Khaini |  |  |  |  |
| Daily/Weekly | 29 | 10.0 | 16 | 3.9 |
| No use | 261 | 90.0 | 393 | 96.1 |
| Tobacco with slaked lime |  |  |  |  |
| Daily/Weekly | 75 | 25.9 | 149 | 36.4 |
| No use | 215 | 74.1 | 260 | 63.6 |
| Gutka |  |  |  |  |
| Daily/Weekly | 44 | 15.2 | 60 | 14.7 |
| No use | 246 | 84.8 | 349 | 85.3 |
| Mava |  |  |  |  |
| Daily/Weekly | 32 | 11.0 | 14 | 3.4 |
| No use | 258 | 89.0 | 395 | 96.6 |
| Mishri |  |  |  |  |
| Daily/Weekly | 58 | 20.0 | 65 | 15.9 |
| No use | 232 | 80.0 | 344 | 84.1 |
| Pan masala with tobacco |  |  |  |  |
| Daily/Weekly | 12 | 4.1 | 7 | 1.7 |
| No use | 278 | 95.9 | 402 | 98.3 |
| Snuff |  |  |  |  |
| Daily/Weekly | 5 | 1.7 | --. | -- |
| No use | 285 | 98.3 | 409 | 100.0 |
| Other smokeless tobacco |  |  |  |  |
| Daily/Weekly | 28 | 9.7 | 72 | 17.6 |
| No use | 262 | 90.3 | 337 | 82.4 |
