# Supplementary material for: Post-discharge tobacco abstinence in a Mumbai hospital after implementation of tobacco cessation counseling: A pragmatic evaluation of the LifeFirst program
Source: PLoS One. 2024 Nov 12;19(11):e0312319. doi: 10.1371/journal.pone.0312319 (PMC11556754; doi:10.1371/journal.pone.0312319)
Supplement: S3 Table — (DOCX) [file pone.0312319.s004.docx]

**S3 Table.** Outcome availability by follow-up timepoint and respondent

| Outcome  Availability | Pre-implementation of LifeFirst (N=437) | | | | Post-implementation of LifeFirst (N=561) | | | |
| --- | --- | --- | --- | --- | --- | --- | --- | --- |
|  | 7 days  n (%) | 30 days  n (%) | 3 months  n (%) | 6 months  n (%) | 7 days  n (%) | 30 days  n (%) | 3 months  n (%) | 6 months  n (%) |
| Surveys completed | 273 (62.5) | 254 (58.1) | 226 (51.7) | 217 (49.7) | 466 (83.1) | 450 (80.2) | 396 (70.6) | 395 (70.4) |
| by Self** | 192 (70.3) | 187 (73.6) | 155 (68.6) | 180 (82.9) | 401 (86.1) | 393 (87.3) | 352 (88.9) | 344 (87.1) |
| by Proxy | 81 | 67 | 71 | 37 | 65 | 57 | 44 | 51 |
| Continuous abstinence inferred from other follow-up time points* | 65 (14.9) | 76 (17.4) | 92 (21.1) | 75 (17.2) | 55 (9.8) | 57 (10.2) | 79 (14.1) | 61 (10.9) |
| Outcome Missing | 99 (22.7) | 107 (24.5) | 119 (27.2) | 145 (33.2) | 40 (7.1) | 54 (9.6) | 86 (15.3) | 105 (18.7) |

*If a follow-up was missed and respondents reported continuous abstinence at later time points then missing outcome status was inferred as continuous abstinence.

**n (%) indicates number of completed surveys with responses from participant (by Self).
