# Supplementary material for: Post-discharge tobacco abstinence in a Mumbai hospital after implementation of tobacco cessation counseling: A pragmatic evaluation of the LifeFirst program
Source: PLoS One. 2024 Nov 12;19(11):e0312319. doi: 10.1371/journal.pone.0312319 (PMC11556754; doi:10.1371/journal.pone.0312319)
Supplement: S5 Table — (DOCX) [file pone.0312319.s006.docx]

**S5 Table.** Odds of continuous abstinence from all tobacco at 6 months comparing all pre-implementation participants (n=437) vs. those post-implementation participants who accepted LifeFirst counseling (n=490), Total n=927

|  | Odds Ratio | | | p-value | Adjusted Odds Ratio^a^ | | | p-value |
| --- | --- | --- | --- | --- | --- | --- | --- | --- |
|  | Estimate | 95% LCL | 95% UCL |  | Estimate | 95% LCL | 95% UCL |  |
| Post- vs. pre-implementation | **3.00** | **2.14** | **4.19** | **<0.001** | **2.98** | **2.00** | **4.44** | **<0.001** |

Abbreviations: OR=odds ratio, aOR=adjusted odds ratio, UCL=upper confidence limit, LCL=lower confidence limit, FTND-Fagerström test for nicotine dependence, PHQ-2=patient health questionnaire 2 item instrument, GAD-2=generalized anxiety disorder 2 item instrument.

^a^Adjusted for tobacco type, age, sex, education, employment, marital status, FTND (FTND for those who smoke only, FTND SL for those who use smokeless only or the highest of the two scores for dual users), confidence in quitting, motivation to quit, beliefs in harms of tobacco (Composite item of “Do you think tobacco use has harmed your health”, “Is tobacco a cause of the illness you are in the hospital for?” and “Do you think quitting now would improve your health?” with responses categorized as Most: reported *Some* or *A lot* for all three items; Some: reported at least one *Some* or *A lot* in any of the three items; Little: reported *A little bit,* *Not at all,* or *Don’t know/refused* for all three items), Admission diagnosis (tobacco related includes cardiovascular disease, chronic lung disease, or tobacco-related cancer), depression and anxiety symptoms.
