# Supplementary material for: Post-discharge tobacco abstinence in a Mumbai hospital after implementation of tobacco cessation counseling: A pragmatic evaluation of the LifeFirst program
Source: PLoS One. 2024 Nov 12;19(11):e0312319. doi: 10.1371/journal.pone.0312319 (PMC11556754; doi:10.1371/journal.pone.0312319)
Supplement: Attachment — Submitted filename: Review.docx [file pone.0312319.s008.docx]

The authors report the effectiveness of a tobacco counselling program (from hospitalization to 6 months post discharge) in a single hospital in Mumbai (India), using a pre-post study design. They found the intervention effective, with an OR of 2.95.

Strengths of the study include a focus on all forms of tobacco (including smokeless tobacco), a comprehensive intervention with structured counsellor training, and an effort to apply it hospital wide.

The study has several methodological limitations that constrain causal inferences. First, the primary outcome is self-reported, rather than a biochemical measure. Second, the pre-post study design is prone to influence from secular trends and unbalanced covariates (seen in Table 1, for instance in confidence in quitting smoking). In addition, outcome assessors were therefore not blinded to study arm assignment, which may have influenced the results. Third, the post period has more patient contacts (duration, if not frequency) than the pre-period, which makes the study susceptible to the Hawthorne effect. This is particularly important given the self-reported nature of the primary outcome. Fourth, 6 month follow up was only obtained in 50% of patients in the pre-group, and 70% in the post group. The overall rates are low, with wide differences between the two groups. Due to these factors, the OR obtained by the authors, is more likely than not, an overestimate of the causal effect of the intervention. The study should be reported as an observational study, given these limitations, and no reliable causal inferences can be made with regards to the effect of the intervention.

Abstract

Conclusion needs to be reworded, to emphasize limitations of study design. Please remove phrases such as ‘real world evidence’. No causal inference can be made from this study, only associations of unclear causality.

Results

Table 2- I am not quite sure what is this table trying to show. There is the primary outcome reported in the first row, and the subsequent rows show the effect of other variables (such as employment/education) on tobacco cessation rates. This should be in a separate table with its own heading, or the current heading should be amended. The current heading is misleading.

Lines 260-262- ‘Follow-up assessments were completed more frequently in the post-implementation phase than the pre-implementation phase (Supplemental Table C, differences all p<0.001).’ Please provide the actual numbers/proportions in the results (the 6 month follow up rates, 50% vs 70%), and not just the p value. This is a crucial piece of information, and should be in the main text.

Discussion

‘It is also one of few studies to assess the feasibility and effectiveness of a hospital-initiated

tobacco cessation intervention conducted outside a high-income country. Whether the

effectiveness of hospital-based cessation treatment that has been demonstrated in high-income settings translates to LMIC settings is not yet established.’

This is not true, based on the references from the introduction of this very paper. An additional reference is below,

<https://link.springer.com/article/10.1007/s11606-023-08243-y>

Conclusions

Lines 358-260: ‘…the magnitude of the difference after adjusting for a long list of measured

characteristics supports the conclusion that this evidence-based counseling intervention

benefits patients and increases abstinence from all tobacco.’ Extremely flawed methods will provide extremely flawed results. The magnitude of the difference, if anything, is likely a result of the flawed study design. Please take a cautious approach to interpreting the observational data, rather than overemphasizing causal inferences (which really cannot be made).

Other points

‘Real world effectiveness’- Please refrain from using this term, as there is no standard definition, and there is only one world, which is real. Using this term distracts from the methodological weaknesses of the study, which the authors should be more upfront about.
