# Supplementary material for: Post-discharge tobacco abstinence in a Mumbai hospital after implementation of tobacco cessation counseling: A pragmatic evaluation of the LifeFirst program
Source: PLoS One. 2024 Nov 12;19(11):e0312319. doi: 10.1371/journal.pone.0312319 (PMC11556754; doi:10.1371/journal.pone.0312319)
Supplement: Attachment — Submitted filename: Response to reviews PLOS ONE 25JUL2024.docx [file pone.0312319.s009.docx]

Response to reviews:

1. The study has several methodological limitations that constrain causal inferences. First, the primary outcome is self-reported, rather than a biochemical measure.

***We have reworded this limitation for emphasis.***

“While biochemical verification is common in randomized trials, in this observational study of intervention delivery within routine hospital practices, it was not feasible.”

2. Second, the pre-post study design is prone to influence from secular trends and unbalanced covariates (seen in Table 1, for instance in confidence in quitting smoking).

***We emphasize this limitation of our observational design.***

***”***The pre-post design is susceptible to secular trends and imbalance between the pre- and post- groups by both measured and unmeasured confounders that could influence our results.”

3. In addition, outcome assessors were therefore not blinded to study arm assignment, which may have influenced the results.

***Patients were not assigned to study arms in this observational study, but we have added a comment to limitations noting that outcome assessors were aware of pre- or post-group status.***

***“***Self- or proxy-report may be subject to social desirability bias and outcome assessors were aware of participant pre- or post-implementation status.”

4. Third, the post period has more patient contacts (duration, if not frequency) than the pre-period, which makes the study susceptible to the Hawthorne effect. This is particularly important given the self-reported nature of the primary outcome.

***We have added this point to our limitations.***

“The study design does not include a placebo or attention matched control in routine practice in the pre-implementation group.”

5. Fourth, 6 month follow up was only obtained in 50% of patients in the pre-group, and 70% in the post group. The overall rates are low, with wide differences between the two groups. Due to these factors, the OR obtained by the authors, is more likely than not, an overestimate of the causal effect of the intervention. The study should be reported as an observational study, given these limitations, and no reliable causal inferences can be made with regards to the effect of the intervention.

***We agree with this point. It is stated in our “Transparency and registration” section where we noted this is observational, and noted it as well in other places in the manuscript. To further address this reviewer comment and eliminate any implication that we are inferring causality we edited the title and an explicit statement about causal inference.***

***“***This design allows us to measure associations, but does not generate causal inference.”

6. Conclusion needs to be reworded, to emphasize limitations of study design. Please remove phrases such as ‘real world evidence’. No causal inference can be made from this study, only associations of unclear causality.

***We have edited the Abstract conclusions, removed the term ‘real-world’ and reworded to clarify that our study generated measures of association only.***

***“***Post-discharge abstinence was more common after implementation of a multi-session tobacco counseling program for hospitalized patients compared to abstinence among patients hospitalized before implementation. These findings represent observational evidence of a promising association between post-discharge abstinence and a hospital-based tobacco cessation program implemented in routine practice in an LMIC setting.”

7. Results; Table 2- I am not quite sure what is this table trying to show. There is the primary outcome reported in the first row, and the subsequent rows show the effect of other variables (such as employment/education) on tobacco cessation rates. This should be in a separate table with its own heading, or the current heading should be amended. The current heading is misleading.

***We have edited this to table to present the primary outcome. We have edited the footnote to list all of the variables adjusted for in the adjusted model and edited the table title.***

8. Lines 260-262- ‘Follow-up assessments were completed more frequently in the post-implementation phase than the pre-implementation phase (Supplemental Table C, differences all p<0.001).’ Please provide the actual numbers/proportions in the results (the 6 month follow up rates, 50% vs 70%), and not just the p value. This is a crucial piece of information, and should be in the main text.

***We have edited the text and added this information to the main text.***

“The proportion of assessments completed by self or proxy was higher post-implementation at all follow-up timepoints (S3 Table; 1 week: pre 62.5% vs. post 83.1%; 1 month: pre 58.1% vs. post 80.2%; 3 months: pre 51.7% vs. post 70.6%, 6 months: pre 49.7% vs. post 70.4%, differences all p<0.001).”

9. Discussion; ‘It is also one of few studies to assess the feasibility and effectiveness of a hospital-initiated tobacco cessation intervention conducted outside a high-income country. Whether the effectiveness of hospital-based cessation treatment that has been demonstrated in high-income settings translates to LMIC settings is not yet established.’

This is not true, based on the references from the introduction of this very paper. An additional reference is below, https://link.springer.com/article/10.1007/s11606-023-08243-y

***We have edited this statement and cited the suggested reference.***

“It adds to a very limited literature studying hospital-initiated cessation counseling support provided to individuals using smokeless tobacco. It also addresses a key gap in the literature, the implementation of hospital-initiated tobacco cessation interventions in LMIC settings, where interventions are often delivered by research nurses, and not studied in routine clinical practice. Positive results have been demonstrated with medical student delivered counseling. Solutions are needed to address tobacco among hospitalized patients in diverse hospital settings including private and other non-academic settings.”

10. Conclusions; Lines 358-260: ‘…the magnitude of the difference after adjusting for a long list of measured characteristics supports the conclusion that this evidence-based counseling intervention benefits patients and increases abstinence from all tobacco.’ Extremely flawed methods will provide extremely flawed results. The magnitude of the difference, if anything, is likely a result of the flawed study design. Please take a cautious approach to interpreting the observational data, rather than overemphasizing causal inferences (which really cannot be made).

***We worked to better emphasize the many limitations in this observational pre-post study design pointed out by the reviewer. However, we were not attempting to present this as a ‘flawed’ RCT. It is a different study design that allows for measures of association within routine practice workflows rather than a controlled experimental trial context. We edited the manuscript to ensure no claim of causality is included in the text and continue to repeatedly label it as observational. We also reworded the sentence noted above.***

***“***While temporal trends and differences in the pre- and post-implementation samples may contribute to this association in this observational design, the association we measured after adjusting for a long list of characteristics suggests that this evidence-based counseling intervention is a promising model for identifying and assisting people who use tobacco in a hospital setting that is worthy of further studies of its effectiveness and implementation.”

11. Other points; ‘Real world effectiveness’- Please refrain from using this term, as there is no standard definition, and there is only one world, which is real. Using this term distracts from the methodological weaknesses of the study, which the authors should be more upfront about.

***We have removed this term. We have edited the text to expand upon the limitations of this observational study design. We continue to label this study as observational, as we did in the original submission. While we did not explicitly describe a causal association in our original discussion, in response to the reviewer’s feedback we adjusted our wording so as not to not imply causality. We believe the edits prompted by this feedback have improved the clarity of our conclusions. This was not intended to distract. We do think there are strengths to our observational design compared to RCTs. RCTs are experimental, they require study workflows beyond routine clinical practice (e.g. randomization, placebo or attention matched controls) and these factors contribute to challenges in translating promising effects from RCTs into routine clinical practice. Observational studies in routine practice have inherent value, not as ‘flawed’ RCTs but as their own contribution to the literature.***

12. Journal Requirements: When submitting your revision, we need you to address these additional requirements. Please ensure that your manuscript meets PLOS ONE's style requirements, including those for file naming. The PLOS ONE style templates can be found at https://journals.plos.org/plosone/s/file?id=wjVg/PLOSOne_formatting_sample_main_body.pdf and  https://journals.plos.org/plosone/s/file?id=ba62/PLOSOne_formatting_sample_title_authors_affiliations.pdf

***We reviewed style requirements and made edits to file naming.***

13. Thank you for stating the following in the Competing Interests section: "GK has a family financial interest in Dimagi Inc. She was not involved in selection of the data collection platform. GK has received research grants from the National Comprehensive Cancer Network with funding from Astra Zeneca. HG is an employee of the Narotam Sekhsaria Foundation. All other authors declare that they have no conflicts of interest."

Please confirm that this does not alter your adherence to all PLOS ONE policies on sharing data and materials, by including the following statement: ""This does not alter our adherence to  PLOS ONE policies on sharing data and materials.” (as detailed online in our guide for authors http://journals.plos.org/plosone/s/competing-interests).  If there are restrictions on sharing of data and/or materials, please state these. Please note that we cannot proceed with consideration of your article until this information has been declared.

Please include your updated Competing Interests statement in your cover letter; we will change the online submission form on your behalf.

***We have added the updated Competing Interests statement with the detail above to our cover letter.***

14. In the online submission form, you indicated that the datasets generated and/or analysed during the current study are not publicly available due to local institutional standards, but are available from the corresponding author on reasonable request. De-identified data from this study will be made available (as allowable according to institutional IRB standards) by emailing the corresponding author. Analytic code used to conduct the analyses presented in this study are not available in a public archive. They may be available by emailing the corresponding author. Materials used to conduct the study are not publicly available.

All PLOS journals now require all data underlying the findings described in their manuscript to be freely available to other researchers, either 1. In a public repository, 2. Within the manuscript itself, or 3. Uploaded as supplementary information.

This policy applies to all data except where public deposition would breach compliance with the protocol approved by your research ethics board. If your data cannot be made publicly available for ethical or legal reasons (e.g., public availability would compromise patient privacy), please explain your reasons on resubmission and your exemption request will be escalated for approval.

***We have generated a deidentified dataset that is in compliance with ethical requirements and uploaded it with our resubmission.***

15. Your ethics statement should only appear in the Methods section of your manuscript. If your ethics statement is written in any section besides the Methods, please move it to the Methods section and delete it from any other section. Please ensure that your ethics statement is included in your manuscript, as the ethics statement entered into the online submission form will not be published alongside your manuscript.

***We have ensured the ethics statement is included only in the Methods section.***

16. Please include captions for your Supporting Information files at the end of your manuscript, and update any in-text citations to match accordingly. Please see our Supporting Information guidelines for more information: http://journals.plos.org/plosone/s/supporting-information.

***We have added captions and in-text citations using the style noted in the links above.***

17. Please review your reference list to ensure that it is complete and correct. If you have cited papers that have been retracted, please include the rationale for doing so in the manuscript text, or remove these references and replace them with relevant current references. Any changes to the reference list should be mentioned in the rebuttal letter that accompanies your revised manuscript. If you need to cite a retracted article, indicate the article’s retracted status in the References list and also include a citation and full reference for the retraction notice.

***We reviewed our reference list.***
