# Supplementary material for: Post-discharge tobacco abstinence in a Mumbai hospital after implementation of tobacco cessation counseling: A pragmatic evaluation of the LifeFirst program
Source: PLoS One. 2024 Nov 12;19(11):e0312319. doi: 10.1371/journal.pone.0312319 (PMC11556754; doi:10.1371/journal.pone.0312319)
Supplement: Attachment — Submitted filename: Response to review letter LifeFirst.docx [file pone.0312319.s010.docx]

August 29, 2024

Dear Editors,

Please find our point by point response to reviews below. We believe the edits have improved the manuscript and look forward to your review and decision.

Sincerely,

Gina Kruse

Gina Kruse MD, MS, MPH

Visiting Associate Professor, Division of General Internal Medicine

University of Colorado School of Medicine

gina.kruse@cuanschutz.edu

**Response to review:**

Journal: Please review your reference list to ensure that it is complete and correct. If you have cited papers that have been retracted, please include the rationale for doing so in the manuscript text, or remove these references and replace them with relevant current references. Any changes to the reference list should be mentioned in the rebuttal letter that accompanies your revised manuscript. If you need to cite a retracted article, indicate the article’s retracted status in the References list and also include a citation and full reference for the retraction notice.

*We pulled up all cited articles in PubMed and found none of our cited works have been retracted.*

**Reviewers’ comments:**

Reviewer #2: This pre-post study measures tobacco abstinence (including smokeless tobacco) among individuals discharged from a Mumbai hospital after the implementation of cessation counseling (LifeFirst) compared to abstinence among those discharged pre-implementation. Given that most tobacco-related diseases occur in low- and middle-income countries, this study addresses a critical need. In addition, few studies have assessed smoking cessation treatment initiated in the hospital in low- and- middle income settings.

• I would like to know more about LifeFirst Services. Are these services still available at the hospital? LifeFirst does not include recommendations for pharmacotherapy. Is this because of availability?

*We have added additional detail about LifeFirst. Pharmacotherapy was not an integral part of LifeFirst because of limited evidence supporting pharmacotherapy use in smokeless tobacco and the high cost of NRT and prescription pharmacotherapies. The program is currently discontinued in the hospital while it undergoes construction. However, based on the experience described in this study the service was expanded and the model is currently being implemented in six other hospitals in Mumbai.*

**LifeFirst Intervention**

“Pharmacotherapy is not integral to the program due to the high costs of nicotine replacement therapy and prescription pharmacotherapies and because of the large proportion of patients using smokeless only, for whom there is less evidence supporting pharmacotherapy use.”

**Discussion**

*“*Adding *provision* of pharmacotherapy to the counseling intervention should further enhance the effect.”

*and*

“Indeed, while the LifeFirst program is currently discontinued in the Prince Aly Khan Hospital during facilities construction, based on the experiences with LifeFirst described here, it is currently being implemented in six other Mumbai hospitals.”

• The authors have addressed the many limitations inherent in observational studies. In future studies, other designs, such as regression discontinuity or difference-in-difference (DD) approaches could be considered to address some of these limitations.

*Thank you for this suggestion, we will consider this in future studies where an observational design is required.*

• The authors can consider citing the reference below which discusses that for some study designs, such as Quitline studies and observational studies, biochemical validation may not be feasible (Reviewer’s comment #1)

Benowitz NL, Bernert JT, Foulds J, Hecht SS, Jacob P, Jarvis MJ, Joseph A, Oncken C, Piper ME. Biochemical Verification of Tobacco Use and Abstinence: 2019 Update. Nicotine Tob Res. 2020 Jun 12;22(7):1086-1097. doi: 10.1093/ntr/ntz132. PMID: 31570931; PMCID: PMC7882145.

*We have added reference to this study.*
